# Viral and Genomic Drivers of Squamous Cell Neoplasms Arising in the Lacrimal Drainage System

**DOI:** 10.3390/cancers14102558

**Published:** 2022-05-23

**Authors:** Ingvild Ramberg, Filipe Garrett Vieira, Peter Bjerre Toft, Christian von Buchwald, Steffen Heegaard

**Affiliations:** 1 The Eye Pathology Section, Department of Pathology, Copenhagen University Hospital Rigshospitalet, DK-2100 Copenhagen, Denmark; ingvild.margrethe.sellaeg.ramberg@regionh.dk; 2 Department of Ophthalmology, Copenhagen University Hospital Rigshospitalet, DK-2100 Copenhagen, Denmark; peter.bjerre.toft@regionh.dk; 3 Department of Clinical Medicine, Copenhagen University, DK-2100 Copenhagen, Denmark; christian.von.buchwald@regionh.dk; 4 Center for Genomic Medicine, Copenhagen University Hospital Rigshospitalet, DK-2100 Copenhagen, Denmark; filippe.garrett.vieira@regionh.dk; 5 Department of Otorhinolaryngology, Head and Neck Surgery and Audiology, Copenhagen University Hospital Rigshospitalet, DK-2100 Copenhagen, Denmark

**Keywords:** lacrimal drainage system, human papillomavirus, squamous cell carcinoma, papilloma

## Abstract

**Simple Summary:**

Carcinomas arising in the lacrimal drainage system (LDS) are rare but notoriously aggressive tumors, causing substantial morbidity and mortality. The molecular drivers of the disease remain unexplored despite being a prerequisite for identifying targets for future prognostication and therapy. Therefore, we aimed to investigate the genomic aberrations in carcinomas arising in the LDS and correlate the findings to human papillomavirus (HPV) status. By detecting transcriptionally active HPV in 80% of LDS papillomas and 67% of LDS carcinomas, we suggest HPV to be an important contributor to carcinogenesis in this location. Further, the genomic profile of the HPV16-positive carcinomas, with activating mutations in the PI3K-AKT signaling cascade, wildtype status of *TP53*, and p16 overexpression, resembles that of HPV-driven disease at other locations with implications for future therapy.

**Abstract:**

The pathogenesis of squamous cell neoplasms arising in the lacrimal drainage system is poorly understood, and the underlying genomic drivers for disease development remain unexplored. We aimed to investigate the genomic aberrations in carcinomas arising in the LDS and correlate the findings to human papillomavirus (HPV) status. The HPV analysis was performed using HPV DNA PCR, HPV E6/E7 mRNA in-situ hybridization, and p16 immunohistochemistry. The genomic characterization was performed by targeted DNA sequencing of 523 cancer-relevant genes. Patients with LDS papilloma (*n* = 17) and LDS carcinoma (*n* = 15) were included. There was a male predominance (68%) and a median age at diagnosis of 46.0 years (range 27.5–65.5 years) in patients with papilloma and 63.8 years (range 34.0–87.2 years) in patients with carcinoma. Transcriptional activity of the HPV E6/E7 oncogenes was detected in the whole tumor thickness in 12/15 (80%) papillomas (HPV6, 11, 16) and 10/15 (67%) squamous cell carcinomas (SCC) (HPV11: 3/15 (20%) and HPV16: 7/15 (47%)). Pathogenic variants in *PIK3CA*, *FGFR3*, *AKT1*, and *PIK3R1*, wildtype *TP53*, p16 overexpression, and deregulated high-risk E6/E7 transcription characterized the HPV16-positive SCC. The deregulated pattern of HPV E6/E7 expression, correlating with HPV DNA presence and p16 positivity, supports a causal role of HPV in a subset of LDS papillomas and carcinomas. The viral and molecular profile of LDS SCC resembles that of other HPV-driven SCC.

## 1. Introduction

Carcinomas of the lacrimal drainage system (LDS)—hence carcinomas derived from the epithelial lining of the lacrimal canaliculi, lacrimal sac, or nasolacrimal duct—are notoriously aggressive tumors constituting challenges in diagnosis and treatment. Since 1960, approximately 550 cases of LDS carcinomas have been reported in the scientific literature [1]. The rareness of the tumors adds an extra layer of complexity to the handling of these patients. Epiphora is a universal symptom for all diseases affecting the LDS, including malignant tumors. Therefore, the early stages of malignant tumors are often misdiagnosed as dacryocystitis or post-inflammatory stenosis, causing a costly delay in diagnosis, since the advanced-stage disease causes more mutilating surgery and increased requirements of adjuvant chemo- and radiotherapy. To date, neither an American Joint Committee on Cancer (AJCC) tumor staging algorithm nor a consensus on treatment regimens exists.

Besides age, the proposed risk factors for LDS carcinoma include chronic dacryocystitis, human papillomavirus (HPV) infection, and LDS papilloma [1]. The association between HPV and carcinomas of the LDS has previously been addressed in five small series (*n* = 27 cases) [2,3,4,5,6]. A high percentage of these tumors were high-risk HPV positive by HPV DNA PCR (21 out of 27, 78%), whereas transcriptionally active HPV was shown in one study [4,5]. The HPV RNA expression in carcinomas of the LDS has not been further investigated, despite the advantage of separating incidental bystander infections from transcriptionally active ones linked to disease development. In light of the prophylactic HPV vaccines available, investigating the importance of HPV in these tumors is timely. Furthermore, investigations of the underlying genetic aberrations that drive the carcinoma development in this location is missing. Such studies are essential to further our understanding of the tumors’ natural history and for inclusion in basket trials, as well as future prognostication and tailoring of specific treatment regimens. Therefore, we aimed to explore the role of HPV in LDS papilloma and carcinoma and investigate the genomic drivers of LDS carcinomagenesis.

## 2. Materials and Methods

### 2.1. Materials

**Figure 1:**
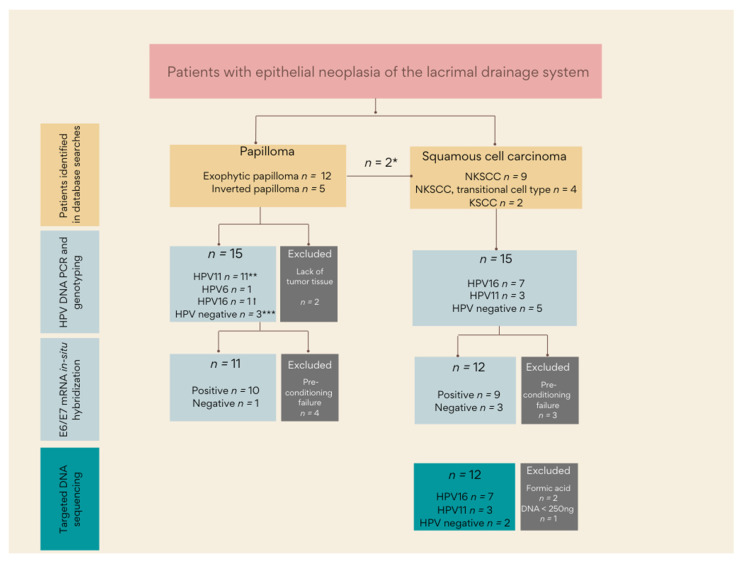
An overview of the workflow for HPV analyses and DNA sequencing. * Malignant transformation occurred in two patients with an LDS papilloma. ** One sample had concurrent infection with HPV11 and 16. *** The HPV-negative papillomas included two exophytic papillomas and one inverted papilloma.

Patients with a histopathological diagnosis of papilloma or a squamous cell carcinoma of the LDS diagnosed in Denmark between January 2000 and January 2020 were included in the study by searching the Danish Pathology Register and manually reviewing the pathology records at the Eye Pathology Section, Department of Pathology, Copenhagen University Hospital Rigshospitalet, Denmark (Figure 1). Two papillomas were included in a previous series from our institution [4]. However, the tumor blocks did not contain enough tumor tissue for further molecular analysis and are thus only included for demographic description in the present series (Figure 1). Clinical data, including histopathological diagnosis, age, gender, size and location of the tumor, treatment, recurrence, and metastasis were extracted from the patients’ medical records when available. The formalin-fixed and paraffin-embedded (FFPE) tumor tissue was retrieved from the pathology departments where the patient was diagnosed. After diagnosis validation, the procedure was as follows: four 5-μm unstained FFPE sections for DNA extraction, sections for HPV E6/E7 mRNA in-situ hybridization, sections for immunohistochemistry, and eventually an HE-stain to confirm the presence of tumor cells. The unstained sections were macroscopically dissected to enrich tumor cells in the downstream analyses.

### 2.2. Methods

The methods for HPV detection, immunohistochemistry, and DNA sequencing have been described previously [7] and are therefore only described briefly here.

#### 2.2.1. DNA Extraction

The Gene Read DNA FFPE kit (#180134; Qiagen, Hilden, Germany) was used for DNA extraction using the QIAcube (Qiagen). DNA quantification was performed on a Qubit DNA HS assay kit (Invitrogen, Thermo Fisher Scientific, Waltham, MA, USA).

#### 2.2.2. HPV DNA PCR and HPV Genotyping

A PCR of the housekeeping gene GAPDH using the GAPDH-a/GAPDH-b primers was performed in all cases to ensure the integrity of the DNA. We expected a 200 base pairs amplicon size for GAPDH. HPV DNA PCR was performed using the GP5+/6+ primers that target a conserved region of the HPV *L1* gene with an expected amplicon size of 150 base pairs. The amplicons, negative controls (containing H_2_0), and positive controls (containing a pool of HPV-positive tumors) were visualized by gel electrophoresis. All HPV-positive amplicons were genotyped by sequencing using the KAPA HTP Library Preparation Kit (Kapa Biosystems, Roche Diagnostics, Basel, Switzerland). The NEXTflex-96 DNA Barcodes (Bioo Scientific, Austin, TX, USA) were ligated. DNA quantity was measured by the Qubit dsDNA BR Kit on a Qubit Fluorometer (Invitrogen). The High Sensitivity D5000 kit (Agilent) was used for quality evaluation of the purified library by automated electrophoreses (TapeStation, Agilent). Sequencing was performed on a MiSeq (Illumina, San Diego, CA, USA). The HPV reference genomes (from the papillomavirus database “PaVe” at https://pave.niaid.nih.gov/ (accessed on 1 March 2022)) were used to map the reads.

#### 2.2.3. HPV E6/E7 mRNA In-Situ Hybridization

The evaluation of the transcriptional activity of the HPV oncogenes E6 and E7 was performed using mRNA in-situ hybridization following the same procedure as previously described [8]. We used the RNAscope vs. Reagent Kit (Advanced Cell Diagnostics, Newark, CA, USA) with the high-risk HPV16 cocktail probe (HPV16, 18, 26, 31, 33, 35, 39, 45, 51, 52, 53, 56, 58, 59, 66, 68, 73, and 82) and the low-risk HPV6 probe (HPV6, 11, 40, 42, 43, and 44).

#### 2.2.4. Immunohistochemistry

All specimens were evaluated for pan-cytokeratin (CK-AECAM) and p16 expression. We performed immunohistochemical evaluations on 4-µm-thick sections using the following clones: CK-AECAM (CM162C; Biocare Medical, Pacheco, CA, USA) and p16 (E6H4; Roche) Staining for CK-AECAM expression was performed on a Dako Autostainer Link 48 (Agilent) using the EnVision Flex+ Detection Kit (K8002, Agilent). Staining for p16 expression was performed on a Ventana Benchmark Ultra (Ventana Medical Systems, Roche, Oro Valley, AZ, USA) using the UltraView/Optiview detection kit (760-500/760-700) following the manufacturer’s instructions. 

p16 expression is used as a phenotypic marker of the HPV E7-mediated degradation of the retinoblastoma protein, thereby acting as a surrogate marker for HPV E6/E7-transforming HPV infections. p16 evaluation was performed prior to HPV analyses and thereby blinded for HPV status. Cytoplasmic and nuclear staining in ≥75% of the cells were considered p16 positive.

#### 2.2.5. DNA Sequencing

The quality of the sequencing libraries quality decreased below the input level of 250 ng, whereas we only experienced minor quality differences between 250 ng and 500 ng DNA input. The input limit was therefore set to 250 ng to include as many samples as possible. Thirteen out of 16 carcinoma samples were eligible for DNA sequencing. For library preparation, we used the TruSight Oncology 500 (TSO500) preparation kit (Illumina, CA, USA) following the TSO500 reference guide (Illumina, #1000000067621 at https://support.illumina.com/downloads/trusight-oncology-500-reference-guide 1000000067621.html (accessed on 1 March 2022)). The TSO500 panel covers 523 cancer-related genes (Table S1). The NovaSeq 6000 platform (Illumina) was used for paired-end sequencing (2 × 150 base pairs) following the manufacturer’s protocol.

#### 2.2.6. Bioinformatic Analysis and Annotation

The bcl2fastq v2.16.0.10 (Illumina) was used to convert raw sequencing data to FASTQ files, followed by a quality assessment of the reads by the FASTQC v.0.11.85 [9]. The reads were trimmed with BBduk v.38.26 and mapped to hg19 using the BWA-MEM v.0.7.12. A quality assessment of the alignment was performed with Mosdepth v.02.4 [10]. The Mutect2 from the Genome Analysis Toolkit (GATK) (Broad Institute, Cambridge, MA, USA) v.4.1.0.0. was used for variant calling following their best practices for tumor-only short somatic variant discovery [11]. The Genome Aggregation Database v. 2.1.1. was used as the germline source [12]. Customized variant annotation was performed using Ingenuity Variant Analysis (Qiagen Bioinformatics, Redwood City, CA, USA, Table S2). Variants classified as “benign” or “likely benign” were excluded, whereas samples classified as “pathogenic,” “likely pathogenic,” or “uncertain” were looked up in the cbioportal.org database to evaluate the possible functional impact of the variant [13,14]. Variants classified as “uncertain” that were not previously reported in the literature were not annotated. The Integrative Genomics Viewer v.2.5.2 was used for visual inspection of all annotated variants to evaluate the variants in the context of the adjacent reads.

#### 2.2.7. Statistical Analysis

The statistical analyses were performed using the statistical software R for Windows version 3.6.1. (R Foundation, Vienna, Austria). Average values were described as the median value (range), and an unpaired t-test was applied to compare continuous variables. The progression-free survival probability, defined as time to recurrence, lymph node or distant metastasis, or death, whichever occurred first, was illustrated by a Kaplan–Meier curve. Only descriptive statistics were applied due to the limited sample size. A *p*-value of <0.05 was considered statistically significant.

## 3. Results

### 3.1. Clinical Characteristics

A total of 30 patients were identified in the database searches; 17 patients with a lacrimal drainage system papilloma (exophytic squamous papilloma (*n* = 12) and inverted papilloma (IP) (*n* = 5)) and 13 patients diagnosed with a carcinoma (SCC (*n* = 10) and NKSCC (*n* = 3)). Two metachronous carcinoma (NKSCC *n* = 1, SCC *n* = 1) evolved from papillomas, and the total number of carcinomas in our cohort was therefore 15. The base line characteristics are summarized in Table 1. Both groups had a male predominance, with 13 out of 17 (76%) males in patients with a papilloma and 10 out of 15 (67%) males among patients with a carcinoma. Patients with a papilloma were significantly younger at the time of diagnosis (median age 46.0, range 27.5–65.5 years) compared to patients with a carcinoma (median age 63.8, range 34.0–87.2 years), mean difference 12.9 (95% CI 3.0–22.8 years, *p* = 0.012). All tumors originated from the lacrimal sac epithelium, except two papillomas originating from the lacrimal canaliculi.

**Figure 2:**
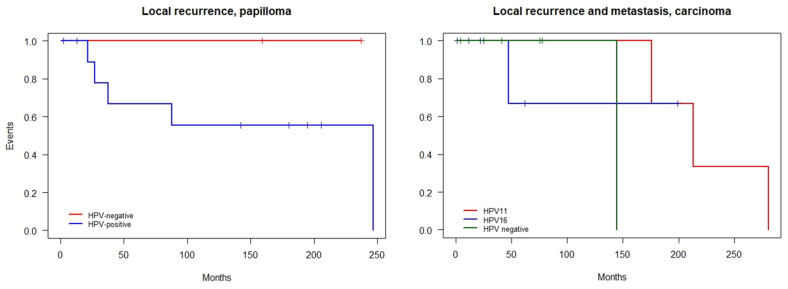
Kaplan–Meier plots illustrating the progression-free survival (PFS) in patients with squamous cell papilloma and carcinoma of the lacrimal drainage system based on human papillomavirus (HPV)-status.

All patients with an LDS carcinoma with available information (*n* = 11) reported epiphora as the initial symptom, with a broad range in duration (median 4.5 years, range 0.5–10 years). Six patients (55%) further reported signs of dacryocystitis with redness, swelling of the medial canthus and palpebra, tenderness, and/or purulent discharge. A medial canthal mass, ulceration, and perforation of the skin were reported in four patients (36%). At diagnosis, six (40%) carcinoma patients had bone invasion and two (13%) had perineural invasion. Ten out of fourteen (71%) had positive surgical margins and were treated with adjuvant radiotherapy, except in one case. Orbital exenteration was performed in two patients. Metastases were reported in three cases (cervical and parotid lymph nodes and extranodular soft tissue on the neck). All three patients had local recurrence before developing metastases. The median progression-free survival was 34.1 months (95% CI 10.4—NA months, range 21.5–88.0) among the papillomas and 22.7 months (95% CI, range 4.6–121.1 months) among the carcinomas (Figure 2). The median follow-up was 61.8 months (range 1.3–280.6).

### 3.2. Human Papillomavirus in LDS Papillomas and Squamous Cell Carcinomas

A total of 30 out of 32 primary tumors were available for HPV DNA PCR (Figure 1). Using HPV DNA PCR, 12 out of 15 (80%, HPV6, 11, 16) of the papillomas and 10 out of 15 (7 out of 15 (47%, HPV16) and 3 out of 15 (20%, HPV11)) of the carcinomas were HPV positive (Table 2).

**Figure 3:**
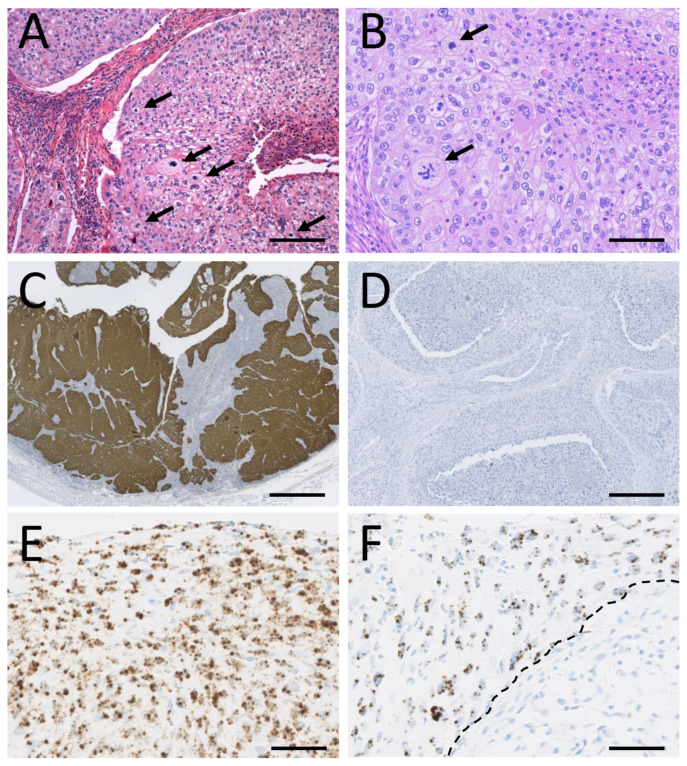
(**A**) An HPV16-positive non-keratinizing squamous cell carcinoma of the lacrimal sac consisting of highly pleomorphic tumor cells with eosinophilic cytoplasm and hyperchromatic nuclei. Numerous aberrant mitoses (arrows) are present (Hematoxylin and eosin (HE), scale bar = 150 µm). (**B**) A pleomorphic HPV11-positive non-keratinizing squamous cell carcinoma with transitional cell morphology of the lacrimal sac with prominent nucleoli and aberrant mitoses (arrows) (HE, scale bar = 100 µm). (**C**) The same tumor as in A, expressing the tumor suppressor p16—a surrogate marker of high-risk HPV infection (p16 immunohistochemistry (IHC), scale bar = 400 µm). (**D**) The low-risk HPV11-associated carcinoma in B does not express p16 (p16 IHC, scale bar = 300 µm. (**E**) Deregulated expression of high-risk HPV E6/E7 with E6/E7 expression in the superficial epithelial layers (chromogenic in-situ hybridization with high-risk HPV E6/E7 mRNA cocktail probe, scale bar = 100 µm) in the same tumor as illustrated in A. By sequencing, the tumor was HPV16-positive. (**F**) Deregulated expression of low-risk E6/E7 transcripts in the same tumor as illustrated in B (chromogenic in-situ hybridization with low-risk HPV E6/E7 mRNA cocktail probe, scale bar = 100 µm) confirmed HPV11-positive by sequencing. Note the sharp demarcation (dashed line) in viral expression between the tumor tissue in the top and the surrounding stroma below.

All recurrences from an HPV-positive primary tumor were also HPV-positive. HPV positivity was confirmed with mRNA ISH, which yielded positive results in all HPV PCR positive primary tumors available for analysis (19 out of 22) and was consistently negative in all HPV DNA-negative cases available (4 out of 8). A deregulated expression pattern of HPV E6/E7—hence expressed throughout the epithelium in both benign and malignant tumors (Figure 3). All HPV16-positive cases expressed p16, yielding a sensitivity of 100% to predict the presence of high-risk HPV. Two high-risk HPV-negative carcinomas (one HPV11 and one HPV negative) and three low-risk papillomas also expressed p16, thereby yielding a specificity of 77%.

### 3.3. Genomic Aberrations in LDS Squamous Cell Carcinoma

**Figure 4:**
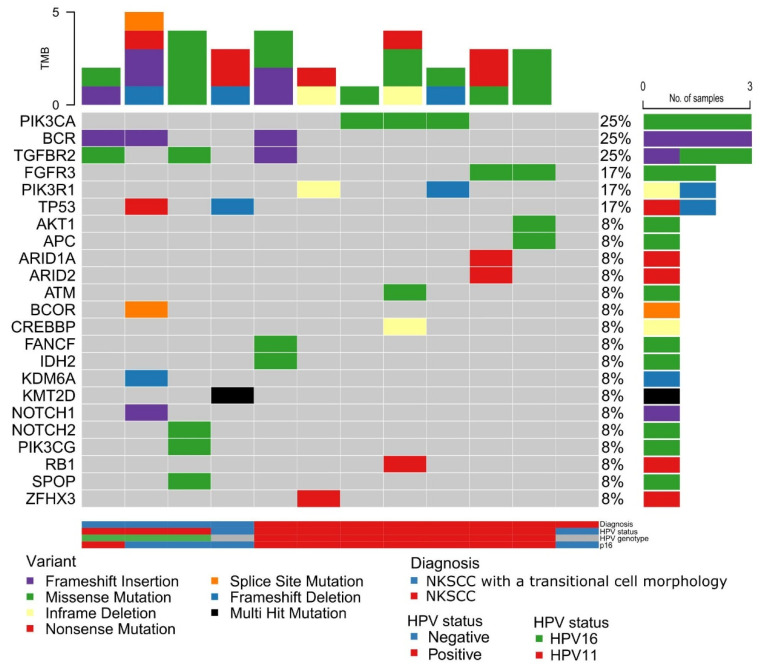
HPV status and somatic mutations in squamous cell carcinomas of the lacrimal drainage system. Six out of seven HPV16-positive SCC harbored mutations in the PI3K-AKT signaling pathway.

Twelve SCCs were available for DNA sequencing, including four carcinomas with transitional cell morphology (Figure 4). The median coverage was 633× (range 251–1067), with 98.9% of the reads above 50×. On average, 3 (range 0–6) pathogenic or likely pathogenic variants were annotated in each sample (Table S3).

*PIK3CA* (p.E542K, p.E545K, p.R115L), *BCR* (p.V1094fs*17), *TGFBR2* (p.A275E, p.K277N), *FGFR3* (p.S249C), *PIK3R1* (p.N673fs*18, c.174635_1748del), and *TP53* (p.Q331*, p.S37fs*7) were altered in more than one case. HPV16-positive cases (*n* = 7, 58%) were characterized by pathogenic variants in *PIK3CA* (3 out of 7), *FGFR3* (2 out of 7), *PIK3R1* (2 out of 7), *AKT1* (1 out of 7), and wildtype *TP53* (all cases). Only two HPV-negative carcinomas were available for analysis. The first harbored a likely pathogenic variant in *TP53* (p.S37fs*7) and a double-hit, truncating variant in *KMT2D* (p.E81* and p.S831*), whereas no somatic pathogenic or likely pathogenic variants were detected in the second HPV-negative sample.

## 4. Discussion

We present the clinical, histopathological, and genetic features of 32 consecutive patients with papilloma and carcinoma of the lacrimal drainage system in a Danish nationwide cohort. The most important findings were: (1) Deregulated transcription of HPV E6/E7 is highly prevalent in both papillomas and squamous cell carcinomas—supporting a causal role of HPV in these tumors, and (2) HPV16-positive LDS SCC has a genomic profile resembling their HPV-positive counterparts of other locations.

The papillomas and the NKSCC with a transitional cell morphology harbored the low-risk genotypes HPV6 and 11, in one case co-existing with the high-risk genotype HPV16. The detection of transcriptionally active low-risk HPV in the exophytic LDS papillomas is in line with previous studies [4], and it is generally accepted that these low-risk genotypes drive the exophytic papillomagenesis in various locations [15,16,17]. Inverted papillomas (IPs) arising in the LDS and the adjacent sinonasal mucosa are associated with aggressive clinical behavior with frequent recurrences and a 5–15% risk of malignant transformation [18,19]. A recent systematic review and meta-analysis concluded that HPV may contribute to the malignant transformation of sinonasal IP, although there is still considerable controversy on this topic [16,20]. HPV has been reported in 50% of LDS IPs (Table S4), and the transcription of viral oncogenes reported in the present study indicates that HPV is related to the papillomagenesis in a subset of these tumors [6,19,21]. 

Despite the designation as a “low-risk” genotype, recent studies support that HPV11 may contribute to malignant transformation [4,22,23] and that the pathogenicity of HPV depends not only on the viral genotype but also on tropism and the host’s immune surveillance [24]. The expression of HPV11 E6/E7 in carcinoma ex-papilloma and carcinomas in the present cohort, and the HPV11 genotypes in general, deserves further investigation to clarify its possible impact on malignant transformation. Although the present study is underpowered to investigate potential prognostic biomarkers in LDS papillomas, it substantiates that LDS papillomas have high recurrence rates and harbor the potential for malignant transformation.

In contrast to the HPV11-positive carcinomas, which all had a non-keratinizing transitional cell morphology, all HPV16-positive carcinomas were non-keratinizing SCC. The oncogenic properties of HPV16, mainly related to the effects of the E6/E7 oncogenes, are well-documented and are an established cause of carcinoma development in the anogenital and head and neck region [25,26]. The combined and maintained expression of E6/E7 can enable all the “cancer hallmarks” in the tissues where they are being expressed [25,27,28]. Though productive HPV infections express low levels of E6/E7 in the basal and parabasal layers, the abortive and transforming infections have a deregulated E6/E7 pattern with full-thickness viral expression [29]. The consistent deregulated HPV16 E6/E7 mRNA pattern in the LDS SCC combined with the expression of p16 in the present series supports a causal role of HPV16 in these tumors.

The HPV16-positive SCCs exhibited a homogeneous molecular profile and seem to be driven by PI3K/AKT signaling pathway activation due to activating, pathogenic variants in *PIK3CA*, *FGFR3*, and *AKT1*, and loss-of-function variants in *PIK3R1* (Figure S1). The gain-of-function variants p.E542K and p.E545K in *PIK3CA* are well-characterized drivers that are frequently altered in HPV-driven SCC of other anatomic locations such as the uterine cervix [30], oropharynx [31], and conjunctiva [7] and are often a result of apolipoprotein B mRNA-editing enzyme, catalytic polypeptide-like (APOBEC)-induced carcinogenesis [30]. Similarly, pathogenic *FGFR3* variants are frequently detected in HPV-positive oropharyngeal SCC, which is associated with a worse prognosis [31]. Furthermore, two cases harbored inactivating variants in *PIK3R1*, leading to the loss of suppression of PI3K, and one sample harbored a pathogenic, activating variant in *AKT1*—coding an oncoprotein that acts downstream to PI3K. In total, 86% of the HPV16-positive tumors (6 out of 7) had activating or loss-of-suppressor variants affecting the PI3K-AKT signaling pathway that causes signaling related to cell growth, cell-cycle progression, and survival, giving rise to a selective growth advantage. All HPV16-positive carcinomas were *TP53* wildtype, a general marker of HPV-positive carcinomas across sites. Since the HPV E6 targets p53 for degradation, a gene-level loss-of-function of *TP53* is not necessary for a functional blockage of p53. Thereby, the genomic profile of HPV16-positive LDS SCC resembles that of other HPV-positive head and neck SCC (HNSCC) [32]. All HPV16-positive LDS carcinomas in the present study were *EGFR* wild-type, supporting the mutual exclusivity between high-risk HPV positivity and *EGFR* mutation observed in the SCC of the neighboring sinonasal cavities [33,34]. Moreover, two out of four HPV-negative and HPV11-positive LDS carcinomas harbored pathogenic *TP53* variants, similar to the keratinizing and HPV-negative sinonasal SCC [34].

### Limitations

The present study was limited by the rarity of the tumors and the formic acid preparation for bone decalcification, which meant only a small number of included samples. This reduced the robustness of our findings and the prognostic role of HPV in these tumors could not be assessed. Furthermore, the lack of normal control tissue to filter germline variants forced us to apply stringent filter criteria in the variant call to avoid spurious calls. All materials used for molecular analyses was formalin-fixed and paraffin embedded, causing potential artifacts in the molecular analyses. A future prospective setup could overcome these challenges. On the other hand, our study is strengthened by the multimodal approach for HPV testing, the deep coverage achieved in the DNA sequencing, and the nationwide setup including consecutive cases with a complete follow-up due to national patient registries.

## 5. Conclusions

We present the largest series to date investigating the transcriptional activity of HPV in LDS papilloma and carcinoma and the correlating histopathological and genomic alterations in a consecutive, nationwide cohort. HPV16-positive LDS SCCs harbor a molecular profile resembling other HPV16-positive carcinomas with p16 expression, wildtype status of *TP53*, frequent pathogenic variants in *PIK3CA* and *FGFR3*, and expression of HPV16 E6/E7, supporting a causal role of HPV16 in these carcinomas. The similarity across sites may be valuable for the future treatment of these rare carcinomas where large studies are hardly feasible and for inclusion in basket trials. Substantial efforts are put into trials of HPV-positive HNSCC, and these results may be important for future patients with HPV16-positive LDS carcinomas.

## Figures and Tables

**Table 1:** Baseline characteristics of patients with a squamous cell carcinoma of the lacrimal drainage system (LDS) in a Danish cohort. HPV; human papillomavirus, M; male, F; female, NKSCC; non-keratinizing squamous cell carcinoma, KSCC; keratinizing squamous cell carcinoma, RT; radiotherapy. * Several patients had multifocal disease. ** Data was missing in some patients.

	HPV11-Positive Carcinoma (*n* = 3)	HPV-16 Positive Carcinoma (*n* = 7)	HPV-Negative Carcinoma (*n* = 5)	Total (*n* = 15)
**Age (years), median (range)**	46 (34–47)	67 (59–82)	52 (49–87)	64 (34–87)
**Gender**	1F/2M	2F/5M	3F/2M	6F/9M
**Previous history of LDS disease, *n* (%)**	2 (67)	1 (14)	2 (40)	5 (33)
**Diagnosis**				
NKSCC, *n* (%)	3 (100)	7 (100)	3 (60)	13 (87)
KSCC, *n* (%)	0 (0)	0 (0)	2 (40)	2 (13)
Local invasion other than nasolacrimal duct, *n* (%) *				
Nasolacrimal duct	1 (50)	3 (42)	0 (0)	4 (31)
Orbit	1 (50)	3 (42)	2 (59)	6 (56)
Ethmoid cells	0 (0)	2 (29)	1 (25)	3 (23)
Maxillary sinus	1 (50)	2 (29)	1 (25)	4 (31)
Nasal cavity	0 (0)	0 (0)	1 (25)	1 (8)
Eyelids	0 (0)	1 (14)	1 (25)	2 (15)
Lacrimal bone	0 (0)	1 (14)	0 (0)	1 (8)
Positive surgical margins **	3 (100)	3 (43)	4 (100)	10 (71)
Adjuvant therapy, *n* (%) **				
Radiotherapy	2 (100)	3 (60)	3 (100)	8 (80)
Cisplatin	0 (0)	0 (0)	1 (33)	1 (10)
**Local recurrence, *n* (%)**	2 (67)	0 (0)	0 (0)	2 (13)
**Metastasis, *n* (%)**				
Lymph nodes	2 (67)	1 (14)	0 (0)	3 (20)
Extranodal tissue	0 (0)	1 (14)	0 (0)	1 (7)
**Follow-up (months), median (range)**	213.2 (175.6–280.6)	25.1 (1.3–199.3)	75.3 (4.3–144.7)	75.3 (1.3–280.6)

**Table 2:** Human papillomavirus status of papillomas and carcinomas of the lacrimal drainage system in a Danish cohort. CI, confidence intervals; HPV, human papillomavirus; LDS, lacrimal drainage system; NA, not applicable; OR, odds ratio.

	LDS Papilloma	LDS Carcinoma
Number of cases	17	15
Previous or concomitant HPV-related head-and-neck neoplasm, *n* (%)	7/17 (41)	4/15 (27)
HPV-positivity (DNA and mRNA), *n* (%)	12/15 (80)	10/15 (67)
HPV genotypes	HPV6, 11, 16	HPV11, 16
P16 positivity, *n* (%)	4/15 (27)	9/15 (60)

## Data Availability

The data will be available from the European Variation Archive at https://www.ebi.ac.uk/eva/ reference number PRJEB47801.

## Supplementary Materials

The following are available online at https://www.mdpi.com/article/10.3390/cancers14102558/s1, Figure S1: A simplified illustration of the PI3K-signalling cascade, Table S1: The 523 genes included in the TruSight Oncology (TSO500) Gene Panel (Illumina, San Diego, CA, USA), Table S2: Ingenuity Variant Analysis filtering criteria, Table S3: Annotated variants, Table S4: Studies reporting on Human papillomavirus (HPV) in inverted papillomas of the lacrimal drainage system.
