# Supplementary material for: Viral and Genomic Drivers of Squamous Cell Neoplasms Arising in the Lacrimal Drainage System
Source: Cancers (Basel). 2022 May 23;14(10):2558. doi: 10.3390/cancers14102558 (PMC9140041; doi:10.3390/cancers14102558)

Viral and genomic drivers of epithelial neoplasms arising in the lacrimal drainage system

Supplementary Material

Supplementary Legends

Supplementary Table 1: The 523 genes included in the TruSight Oncology (TSO500) Gene Panel (Illumina, San Diego, CA, USA).
Supplementary Table 2: Ingenuity Variant Analysis filtering criteria (based on the TSO500 default filtering)
Supplementary Table 3: All annotated variants.
Supplementary Table 4: Studies reporting on Human papillomavirus (HPV) in inverted papillomas of the lacrimal drainage system.
Supplementary Figure 1: The PI3K-signalling cascade, simplified. Created with BioRender.com.

Supplementary Tables

Supplementary Table 1

ABL1	CDKN2C	FAS	HOXB13	MRE11A	PPARG	SOX2
ABL2	CEBPA	FAT1	HRAS	MSH2	PPM1D	SOX9
ACVR1	CENPA	FBXW7	HSD3B1	MSH3	PPP2R1A	SPEN
ACVR1B	CHD2	FGF1	HSP90AA1	MSH6	PPP2R2A	SPOP
AKT1	CHD4	FGF10	ICOSLG	MST1	PPP6C	SPTA1
AKT2	CHEK1	FGF14	ID3	MST1R	PRDM1	SRC
AKT3	CHEK2	FGF19	IDH1	MTOR	PREX2	SRSF2
ALK	CIC	FGF2	IDH2	MUTYH	PRKAR1A	STAG1
ALOX12B	CREBBP	FGF23	IFNGR1	MYB	PRKCI	STAG2
ANKRD11	CRKL	FGF3	IGF1	MYC	PRKDC	STAT3
ANKRD26	CRLF2	FGF4	IGF1R	MYCL1	PRSS8	STAT4
APC	CSF1R	FGF5	IGF2	MYCN	PTCH1	STAT5A
AR	CSF3R	FGF6	IKBKE	MYD88	PTEN	STAT5B

ARAF	CSNK1A1	FGF7	IKZF1	MYOD1	PTPN11	STK11
ARFRP1	CTCF	FGF8	IL10	NAB2	PTPRD	STK40
ARID1A	CTLA4	FGF9	IL7R	NBN	PTPRS	SUFU
ARID1B	CTNNA1	FGFR1	INHA	NCOA3	PTPRT	SUZ12
ARID2	CTNNB1	FGFR2	INHBA	NCOR1	QKI	SYK
ARID5B	CUL3	FGFR3	INPP4A	NEGR1	RAB35	TAF1
ASXL1	CUX1	FGFR4	INPP4B	NF1	RAC1	TBX3
ASXL2	CXCR4	FH	INSR	NF2	RAD21	TCEB1
ATM	CYLD	FLCN	IRF2	NFE2L2	RAD50	TCF3
ATR	DAXX	FLI1	IRF4	NFKBIA	RAD51	TCF7L2
ATRX	DCUN1D1	FLT1	IRS1	NKX2-1	RAD51B	TERC
AURKA	DDR2	FLT3	IRS2	NKX3-1	RAD51C	TERT
AURKB	DDX41	FLT4	JAK1	NOTCH1	RAD51D	TET1
AXIN1	DHX15	FOXA1	JAK2	NOTCH2	RAD52	TET2
AXIN2	DICER1	FOXL2	JAK3	NOTCH3	RAD54L	TFE3
AXL	DIS3	FOXO1	JUN	NOTCH4	RAF1	TFRC
B2M	DNAJB1	FOXP1	KAT6A	NPM1	RANBP2	TGFBR1
BAP1	DNMT1	FRS2	KDM5A	NRAS	RARA	TGFBR2
BARD1	DNMT3A	FUBP1	KDM5C	NRG1	RASA1	TMEM127
BBC3	DNMT3B	FYN	KDM6A	NSD1	RB1	TMPRSS2
BCL10	DOT1L	GABRA6	KDR	NTRK1	RBM10	TNFAIP3
BCL2	E2F3	GATA1	KEAP1	NTRK2	RECQL4	TNFRSF14
BCL2L1	EED	GATA2	KEL	NTRK3	REL	TOP1
BCL2L11	EGFL7	GATA3	KIF5B	NUP93	RET	TOP2A
BCL2L2	EGFR	GATA4	KIT	NUTM1	RFWD2	TP53
BCL6	EIF1AX	GATA6	KLF4	PAK1	RHEB	TP63
BCOR	EIF4A2	GEN1	KLHL6	PAK3	RHOA	TRAF2
BCORL1	EIF4E	GID4	KMT2B	PAK7	RICTOR	TRAF7
BCR	EML4	GLI1	KMT2C	PALB2	RIT1	TSC1
BIRC3	EP300	GNA11	KMT2D	PARK2	RNF43	TSC2
BLM	EPCAM	GNA13	KRAS	PARP1	ROS1	TSHR
BMPR1A	EPHA3	GNAQ	LAMP1	PAX3	RPS6KA4	U2AF1

BRAF	EPHA5	GNAS	LATS1	PAX5	RPS6KB1	VEGFA
BRCA1	EPHA7	GPR124	LATS2	PAX7	RPS6KB2	VHL
BRCA2	EPHB1	GPS2	LMO1	PAX8	RPTOR	VTCN1
BRD4	ERBB2	GREM1	LRP1B	PBRM1	RUNX1	WISP3
BRIP1	ERBB3	GRIN2A	LYN	PDCD1	RUNX1T1	WT1
BTG1	ERBB4	GRM3	LZTR1	PDCD1LG2	RYBP	XIAP
BTK	ERCC1	GSK3B	MAGI2	PDGFRA	SDHA	XPO1
C11orf30	ERCC2	H3F3A	MALT1	PDGFRB	SDHAF2	XRCC2
CALR	ERCC3	H3F3B	MAP2K1	PDK1	SDHB	YAP1
CARD11	ERCC4	H3F3C	MAP2K2	PDPK1	SDHC	YES1
CASP8	ERCC5	HGF	MAP2K4	PGR	SDHD	ZBTB2
CBFB	ERG	HIST1H1C	MAP3K1	PHF6	SETBP1	ZBTB7A
CBL	ERRFI1	HIST1H2BD	MAP3K13	PHOX2B	SETD2	ZFHX3
CCND1	ESR1	HIST1H3A	MAP3K14	PIK3C2B	SF3B1	ZNF217
CCND2	ETS1	HIST1H3B	MAP3K4	PIK3C2G	SH2B3	ZNF703
CCND3	ETV1	HIST1H3C	MAPK1	PIK3C3	SH2D1A	ZRSR2
CCNE1	ETV4	HIST1H3D	MAPK3	PIK3CA	SHQ1	
CD274	ETV5	HIST1H3E	MAX	PIK3CB	SLIT2	
CD276	ETV6	HIST1H3F	MCL1	PIK3CD	SLX4	
CD74	EWSR1	HIST1H3G	MDC1	PIK3CG	SMAD2	
CD79A	EZH2	HIST1H3H	MDM2	PIK3R1	SMAD3	
CD79B	FAM123B	HIST1H3I	MDM4	PIK3R2	SMAD4	
CDC73	FAM175A	HIST1H3J	MED12	PIK3R3	SMARCA4	
CDH1	FAM46C	HIST2H3A	MEF2B	PIM1	SMARCB1	
CDK12	FANCA	HIST2H3C	MEN1	PLCG2	SMARCD1	
CDK4	FANCC	HIST2H3D	MET	PLK2	SMC1A	
CDK6	FANCD2	HIST3H3	MGA	PMAIP1	SMC3	
CDK8	FANCE	HLA-A	MITF	PMS1	SMO	
CDKN1A	FANCF	HLA-B	MLH1	PMS2	SNCAIP	
CDKN1B	FANCG	HLA-C	MLL	PNRC1	SOCS1	
CDKN2A	FANCI	HNF1A	MLLT3	POLD1	SOX10	
CDKN2B	FANCL	HNRNPK	MPL	POLE	SOX17	

Supplementary Table 2

Filter	Inclusion criteria
Confidence	Call quality > 20
	Variant passed upstream pipeline filtering
	Read depth > 100
	Allele fraction > 10
	Outside top 5% most exonically variable 100base windows in healthy public genomes
	Outside top 1% most exonically variable genes in healthy public genomes (100Genomes)
Common variants	<1% of all in gnomAD
	<1% of all in ExAc
	<1% of all in NHLBI ESP exomes
	<1% of all in the 1000 Genomes Project
Predicted deleterious	Pathogenic variants (ACMG Guidelines classification)
	Likely pathogenic variants (ACMG Guidelines classification)
	Uncertain significance (ACMG Guidelines classification)
	Variants listed in HGMD, ClinVar, CentoMD
	Variants established in the literature

Supplementary Table 3

Sample	HPV DNA	HPV E6/E7	p16	HPV genotype	Diagnosis	Read depth	Chromosome location	Gene	AA Change	Transcript	Nucleotide change	Translational impact	Variant allele	Computed classification*
1	Positive	Positive	Positive	HPV11	NKSCC	229	chr3:30713499	*TGFBR2*	p.A275E	NM_003242.6	c.824C>A	Gain	61	Uncertain
						181	chr22:23653975	*BCR*	p.V1050fs*17	NM_021574.3	c.3143_3146dupCC	Loss	14	Uncertain
2	Positive	Positive	Negative	HPV11	NKSCC	238	chrX:39913571	*BCOR*	NA	NM_017745.6	c.4640-105_4655delTCCAGCCTGTCATGAATGTATAGGAAAAAATGCCACGTGGCTCTGCCATCATTTTGTTCTCAGCAGTAGATTTTATCTTGTTTTTTTATTTTTCTCTCCCTCCAGATTATTTAAATGACCT	Loss	18	Likely pathogenic
						318	chr17:7576855	*TP53*	p.Q331*	NM_000546.6	c.991C>T	Loss	30	Pathogenic
						231	chr9:139401863	*NOTCH1*	p.N1179fs*4	NM_017617.5	c.3536dupA	Loss	26	Uncertain
						353	chrX:44929416	*KDM6A*	p.N839fs*6	NM_021140.4	c.2517_2518delCA	Loss	24	Likely pathogenic
						236	chr22:23653975	*BCR*	p.V1050fs*17	NM_021574.3	c.3143_3146dupCCGG	Loss	10	Uncertain
3	Positive	Positive	Negative	HPV11	NKSCC	218	chr1:120462032	*NOTCH2*	p.R1895H	NM_024408.4	c.5684G>A	NA	47	Uncertain
						306	chr3:30713506	*TGFBR2*	p.K277N	NM_003242.6	c.831G>T	Gain	63	Uncertain
						147	chr7:106526601	*PIK3CG*	p.F965S	NM_002649.3	c.2894T>C	Loss	53	Uncertain
						214	chr17:47696716	*SPOP*	p.E78K	NM_003563.3	c.232G>A	Loss	26	Likely pathogenic
4	Negative	Negative	Negative	NA	NKSCC	454	chr12:49448470	*KMT2D*	p.E81*	NM_003482.4	c.241G>T	Loss	36	Likely pathogenic
						417	chr12:49444974	*KMT2D*	p.S831*	NM_003482.4	c.2492C>G	Loss	33	Likely pathogenic
						349	chr17:7579579	*TP53*	p.S37fs*7	NM_000546.6	c.108delG	Frameshift	55	Likely pathogenic
5	Positive	Positive	Positive	HPV16	SCC	101	chr3:30691871	*TGFBR2*	p.P129fs*3	NM_003242.6	c.383dupA	Loss	10	Uncertain
						314	chr11:22647208	*FANCF*	p.R50L	NM_022725.4	c.149G>T	NA	25	Uncertain
						1071	chr15:90631914	*IDH2*	p.V147I	NM_002168.4	c.439G>A	NA	61	Uncertain
						654	chr22:23653975	*BCR*	p.V1050fs*17	NM_021574.3	c.3143_3146dupCCGG	Loss	15	Uncertain
6	Positive	Positive	Positive	HPV16	SCC	902	chr5:67591211	*PIK3R1*	NA	NM_181523.3	c.1746-35_1748delAGTTAATGCGTTCTCTTTTCAAAACTGTTTTTCAGGTG	Loss	17	Likely pathogenic
						361	chr16:72822574	*ZFHX3*	p.Q3201*	NM_001164766.2	c.9601C>T	Loss	40	Likely pathogenic

Sample	HPV DNA	HPV E6/E7	p16	HPV genotype	Diagnosis	Read depth	Chromosome location	Gene	AA Change	Transcript	Nucleotide change	Translational impact	Variant allele	Computed classification*
7	Positive	Positive	Positive	HPV16	SCC	140	chr3:178936082	*PIK3CA*	p.E542K	NM_006218.4	c.1624G>A	Gain	36	Pathogenic
8	Positive	Positive	Positive	HPV16	SCC	174	chr3:178936091	*PIK3CA*	p.E545K	NM_006218.4	c.1633G>A	Gain	40	Pathogenic
						131	chr13:49027168	*RB1*	p.R579*	NM_000321.3	c.1735C>T	Loss	21	Pathogenic
						102	chr16:3781324	*CREBBP*	p.S1642del	NM_001079846.1	c.4925_4927delCCT	Loss	25	Pathogenic
						112	chr11:108206607	*ATM*	p.Q2729H	NM_000051.4	c.8187A>T	Loss	21	Likely pathogenic
						142	chr1:16259147	*SPEN*	p.S2139*	NM_015001.3	c.6414_6415delAA	Loss	24	Likely pathogenic
						139	chr1:16257629	*SPEN*	p.K1632fs*28	NM_015001.3	c.4896_4897delAA	Loss	20	Likely pathogenic
9	Positive	Positive	Positive	HPV16	SCC	127	chr3:178916957	*PIK3CA*	p.R115L	NM_006218.4	c.344G>T	Gain	17	Pathogenic
						113	chr5:67593270	*PIK3R1*	p.N673fs*18	NM_181523.3	c.2019_2022delCAAA	Loss	38	Likely pathogenic
10	Positive	Positive	Positive	HPV16	SCC	273	chr4:1803568	*FGFR3*	p.S249C	NM_000142.5	c.746C>G	Gain	37	Pathogenic
						141	chr1:27023696	*ARID1A*	p.Q268*	NM_006015.6	c.802C>T	Loss	72	Likely pathogenic
						619	chr12:46231185	*ARID2*	p.L370*	NM_152641.4	c.1109delT	Loss	33	Likely pathogenic
11	Positive	Positive	Positive	HPV16	SCC	971	chr14:105246551	*AKT1*	p.E17K	NM_005163.2	c.49G>A	Gain	42	Pathogenic
						493	chr4:1803568	*FGFR3*	p.S249C	NM_000142.5	c.746C>G	Gain	12	Pathogenic
						1142	chr5:112175211	*APC*	p.I1307K	NM_000038.6	c.3920T>A	Loss	50	Pathogenic
12**	Negative	Negative	Negative	NA	SCC	NA	NA	NA	NA	NA	NA	NA	NA	NA
13	Negative	Negative	Negative	NA	Adenocarcin	531	chr17:29677208	*NF1*	p.T2423fs*4	NM_000267.3	c.7267dupA	Loss	28	Likely pathogenic
						631	chr17:7577085	*TP53*	p.E285K	NM_000546.6	c.853G>A	Loss	26	Pathogenic

* Computed classification of pathogenicity by Qiagen Interpret, USA, based on: Richards S, Aziz N, Bale S, Bick D, Das S, Gastier-Foster J, Grody WW, Hegde M, Lyon E, Spector E, Voelkerding K, Rehm HL; ACMG Laboratory Quality Assurance Committee. Standards and guidelines for the interpretation of sequence variants: a joint consensus recommendation of the American College of Medical Genetics and Genomics and the Association for Molecular Pathology. Genet Med. 2015 May;17(5):405-24. doi: 10.1038/gim.2015.30. Epub 2015 Mar 5. PMID: 25741868; PMCID: PMC4544753.

Study	N (cases)	Median age (range)	Gender	HPV positive (%)	HPV genotype	HPV detection modality	Recurrence	Malignant transformation
Wang *et al.* 2021[1]	13	57 (43-84)	7M / 6F	2 / 9 (22)	HPV16, 18, 31, 33, 51	Unknown	2 / 13, median follow-up 48 months (range 2 - 241)	4 / 13 (44)
Jones *et al.* 2020[2]	2	NA	NA	2 / 2 (100)	11, 16	HPV DNA PCR	NA	NA
Raemdock *et al.* 2009[3]	1	24	F	1 / 1	Low-risk	HPV DNA ISH	0 / 1 (follow-up 2 years)	0 / 1
Present study	5	52 (45-56)	2M / 3F	3 / 4 (75)	HPV11, 16	HPV DNA PCR, p16, E6/E7 mRNA ISH	2 / 6, median follow up time 96 months (range 2 - 176)	2 / 6 (33)
**Total**	**22**	**55 (24-84)**	**10M / 10F**	**8 / 16 (50)**	**Low-risk: HPV11 High-risk: HPV16,18, 31, 33, 51**		**4 / 20, median follow-up 48 months (range 2-241)**	**6 / 20 (32)**

Supplementary Table 4

1. J. Wang, J. Ford, B. Esmaeli, P. Langer, N. Esmaili, G. J. Griepentrog, et al. *Inverted papilloma of the orbit and nasolacrimal system.* Ophthalmic Plast Reconstr Surg 2021 Vol. 37 Issue 2 Pages 161-167. DOI: 10.1097/iop.0000000000001719.

2. H. Jones, S. Gane, J. Rimmer, K. Cuschieri and V. J. Lund. *HPV may not play a role in all lacrimal transitional cell papilloma.* Rhinology 2020 Vol. 10.4193/Rhin19.283. DOI: 10.4193/Rhin19.283

3. T. Y. Raemdonck, C. M. Van den Broecke, I. Claerhout and C. E. Decock. *Inverted papilloma arising primarily from the lacrimal sac.* Orbit 2009 Vol. 28 Issue 2-3 Pages 181-4. DOI: 10.1080/01676830802692914

Supplementary Figures

Supplementary Figure 1

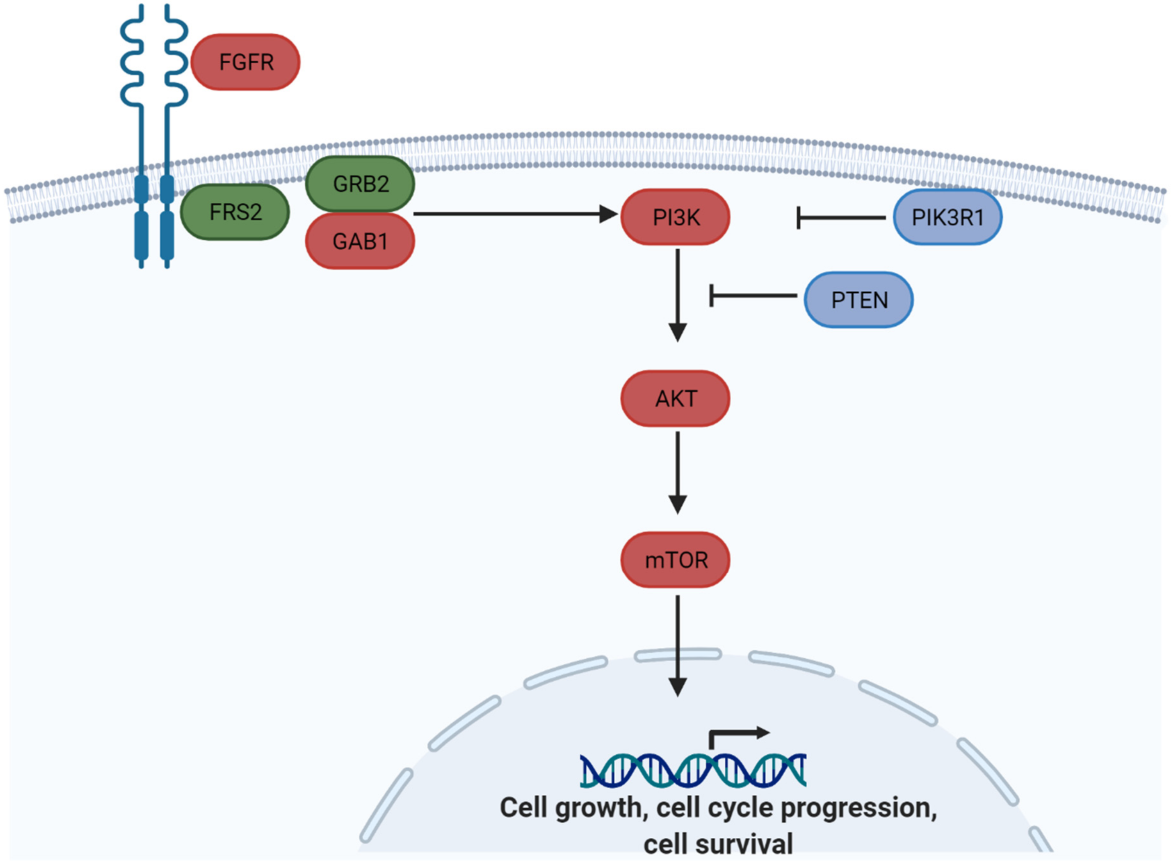

Supplement: Supplementary file 1 [file cancers-14-02558-s001.zip › cancers-1665009-supplementary.pdf]
